# Hybrid quantum-classical neural networks for real-time fault detection in power systems

**DOI:** 10.1371/journal.pone.0349887

**Published:** 2026-06-18

**Authors:** Hina Hashmi, Aman Kumar, Shanu Rakesh Kuttan, Jhankar Moolchandani, Priya Goel, Aseel Smerat, Yaikob Abriham Massebo, Arshad Hashmi

**Affiliations:** 1 Department of Computer Science & Engineering, Moradabad Institute of Technology, Moradabad India; 2 Department of CSE, SET, Manav Rachna International Institute of Research and Studies (Deemed to be University), Faridabad, India; 3 Department of Computer Science and Engineering, Chouksey Engineering College, Bilaspur, Chhattisgarh, India; 4 Department of Computer Science & Engineering, Amity School of Engineering and Technology, Amity University, Gwalior, Madhaya Pradesh, India; 5 Department of Artificial Intelligence and Machine Learning, Manipal University Jaipur, Jaipur, Rajasthan, India; 6 Faculty of Educational Sciences, Al-Ahliyya Amman University, Amman, Jordan‌‌; 7 Department of Biosciences, Saveetha School of Engineering, Saveetha Institute of Medical and Technical Sciences, Chennai, India; 8 Department of English Language and Literature, Gambella University, Gambela, Ethiopia; 9 Department of Information Systems, Faculty of Computing and Information Technology in Rabigh (FCITR), King Abdulaziz University, Jeddah, Saudi Arabia; Maha Bharathi Engineering College, INDIA

## Abstract

As modern power systems continue to be integrated with renewable energy sources, the ability to detect faults in these systems quickly and accurately is becoming more sophisticated. A Hybrid Quantum Classical Neural Network (HQCNN) model is presented in this paper that addresses the real-time fault detection challenge in transmission systems. A classical feature extractor, in this case a 1D CNN, and a quantum circuit are combined by the model, which aids in the classification of faults. Evaluations were conducted on simulated IEEE 14 and 39-bus system and on approximately 800 real PMU fault events. An accuracy of 96.43% on simulated data and 94.74% on real PMU data was achieved by the model, outperforming traditional deep learning models and maintaining detection times under 3 milliseconds. Various kind of fault like SDL (single line to ground), DL (double line), TP (three phase), and high-impedance faults, were correctly classified by the system. In a study in which elements of the model were removed, the quantum layer was found to be very important for improved performance. Issues like hardware limits and quantum noise were also looked at. As for the future, larger PMD data sets will be worked on, model explainability will be improved with hybrid XAI methods, and smaller HQCNN models will be developed for use in substation edge devices.

## 1. Introduction

Power systems and electric cars are being implemented on the modern power grid systems. As they are being implemented, clean energy and decentralized generation are being supported by these changes, which are also creating new issues. From these challenges, one issue is related to the quick and precise detection of faults that need to be ensured. Some other Issues like short circuits, line-to-ground problems, and equipment failures can cause wide-scale outages if not attended in time [1]. These related troubles are often encountered by traditional protection methods that use fixed thresholds values and perform impedance calculations in modern grids, which outputs high variability, noise, and complex network structures. Hence, there is a need to utilize machine learning techniques to overcome these problems. In the case of faults, deep learning has shown improvements in detection, through which complex patterns are learned from the traces of voltage and current signals [2,3]. Some models like CNNs and LSTMs, are used often which are performed very well in classification and time-based analysis [4]. However, large, labeled data sets are usually required by these models, long training times are reported, and a great need for computing power is present, which in turn makes them a hard sell for real-time or edge-based applications [5].Additionally, many of the deep learning models function like “black boxes,” which does not provide much insight into the decision-making process [6,7].

I. Quantum Computing: A New Paradigm

A new way to address complex, high-dimensional learning problems is offered by quantum computing using quantum mechanical effects such as entanglement, superposition, etc. [8]. Significant speedups in data processing are potential achievements of QML(Quantum Machine Learning), especially for tasks like clustering, classification, optimization, etc. [9]. However, major limitations are still present in present quantum hardware that we have known as NISQ devices that includes a fixed count of qubits, imperfect gate fidelity, and high sensitivity to noise [10,11]. Because of these constraints, full practicality for deployment in real-world fault detection systems is not yet achieved by fully quantum models.

II. Hybrid Quantum Classical Neural Networks

To overcome the hardware limitations and still benefiting from its strengths, an HQCNNs (Hybrid Quantum-Classical Neural Networks) is proposed. Classical components such as CNNs (convolutional neural networks) are combined with parameterized quantum circuits (PQCs) to improve feature learning and classification. More accurate data processing is achieved by using the power of both classical and quantum models with current hybrid approach. High accuracy, fast response, and efficient computation are offered by it, making perfect smart grid system to achieve real time fault detection.

III. Objectives and Contributions

An HQCNN(Hybrid Quantum Classical Neural Network) is designed for real-time fault diagnosis in power grid systems is proposed in this study. Quantum computing layers are incorporated into a conventional deep learning architecture by the model, allowing complex patterns in transient electrical signals to be effectively captured while maintaining reasonable computational cost. The primary contributions of the paper are:

- Designing, implementation of an HQCNN for fault classification using voltage and current waveform data.
- Development of a simulation environment using IEEE 14-bus and 39-bus systems to generate labeled fault data.
- Performance benchmarking of the proposed HQCNN model against conventional LSTM, CNN models in terms of and computational efficiency, accuracy, and latency.
- Analysis of quantum circuit configurations and encoding methods to evaluate the trade-offs between model accuracy and quantum resource requirements.

IV. Paper Structuring

The work is drafted by section 1 is itself having the introduction, followed by Section 2, that is dedicated for related work on classical and quantum approaches to fault detection. Next, in section 3, the proposed hybrid model and data processing techniques are elaborated. Following section 4 describes various simulation setup, evaluation process. Finally, section 5 showcases conclusion and discussion of the results.

## 2. Related work

Accurate and real-time fault detection in power systems is regarded as a critical component of intelligent grid operation. Prior research is categorized in this section into four main areas: traditional protection techniques, Quantum Machine Learning applications, classical and deep learning-based fault diagnosis, hybrid quantum classical neural networks, etc.

### 2.1. Conventional fault detection techniques

Traditional methods for fault detection are included by over current protection, distance relays, differential protection, and impedance-based techniques [12,13]. Fixed thresholds are relied upon by these systems and manual configuration for each network topology is often required, which can be error-prone under dynamic grid conditions. To improve fault localization, time-frequency analysis, noise tolerance methods such as the transform using wavelet [14], the S-transform [3], and Hilbert-Huang Transform (HHT) [15] have been introduced. Although better transient feature extraction is offered by these methods, performance still degrades in noisy environments and careful tuning is required. Mathematical morphology [16] and Kalman filtering [8] have been explored by some studies as signal enhancement tools for improving the reliability of traditional detection frameworks. However, rule-based characteristics remained by these approaches and adaptability to grid reconfigurations, or the stochastic nature of RES is often lacking.

### 2.2. Deep learning, machine learning approaches

New avenues for data-driven fault detection have been opened by machine learning (ML). Statistical and spectral features extracted from voltage and current signals have been widely applied using SVMs (Support Vector Machines), Random Forests and Decision Trees [9,17]. This capability is further enhanced by deep learning (DL) through the automatic learning of hierarchical features. Spatial pattern recognition in waveform data has been achieved using CNNs [18], while time dependencies in transient responses have been effectively captured by LSTMs [19]. Hybrid CNN-LSTM architectures have been developed by several researchers to leverage the strengths of both models [20,21]. Anomaly detection and data augmentation in imbalanced fault datasets have also been explored using Autoencoders and Generative Adversarial Networks (GANs) [22,23]. Despite their strong performance, substantial computational power and large training datasets are typically demanded by deep learning models, which limit their feasibility for real-time edge-based applications. Furthermore, challenges in safety-critical domains such as power system protection are posed by the opaque nature of these models. While approaches such as attention mechanisms, explainable AI have been proposed for improving the transparency, uncommon remains their practical deployment in operational systems [24,25].

### 2.3. Power systems using quantum machine learning

QML has been emerged as a useful approach for handling high-dimensional complex problems, including tasks such as classification, regression, and clustering. Potential advantages over classical techniques in specific cases have been shown by several quantum algorithms, including variational quantum classifiers, quantum support vector machines, and quantum kernel methods [26]. Within energy systems, QML has been explored for applications such as load prediction, energy usage analysis, and power flow optimization. An underexplored area remains fault detection, though the feasibility of using quantum algorithms to classify transient waveforms has been demonstrated in initial efforts. Due to the limitations of NISQ devices like qubit decoherence and limited circuit depth, fully quantum models are not yet considered suitable for real-time grid applications [27]. As a result, a shift towards hybrid approaches is being seen in research.

### 2.4. Hybrid quantum classical neural networks

Classical deep learning components are integrated with quantum layers—usually parameterized quantum circuits (PQCs)—in hybrid quantum-classical neural networks (HQCNNs) [28,29]. Quantum-enhanced feature mapping is allowed by these architectures while classical processing is retained for scalability and stability. Circuit-centric quantum classifiers and quantum convolutional networks (QCNNs) have been successfully applied to image recognition, cybersecurity, and finance [30,31]. Hybrid quantum models have also been explored in small-scale power systems for short-term load forecasting and anomaly detection [32].‌‌ This paper aims to fill that gap by designing and evaluating a HQCNN model that classifies fault types based on transient waveform features using a simulation-based dataset from IEEE test systems. Table 1 depicts the summary of various literature Review on Fault Detection in Power Systems.

**Table 1:** Literature Review Summary on Fault Detection in Power Systems.

Conventional Methods				
Ref. No.	Methodology	Dataset Type	Key Contribution	Limitation
[12]	Neural Networks	Simulated	Fast fault detection using neural nets in transmission lines	Fixed architecture, low adaptability
[14]	Wavelet Transform	Simulated	Transient feature extraction for faults	Sensitive to noise, window size selection
[15]	Hilbert-Huang Transform (HHT)	Simulated	Nonlinear signal analysis for transient detection	Computationally expensive
[16]	Mathematical Morphology	Real Fault Logs	Robust noise filtering for transient detection	Limited to binary signal interpretation
[8]	Kalman Filtering	Simulated	Enhanced signal quality in fault detection	Requires accurate system modeling
**Machine Learning Methods**				
[9]	SVM	Simulated	Fault classification from current/voltage signals	Requires hand-crafted features
[33]	Random Forest	Simulated	Ensemble-based robust classification	Less effective with time-series data
[17]	Decision Trees	Real + Simulated	Simple and interpretable fault detection	Low accuracy for complex transients
**Deep Learning Methods**				
[18]	CNN	Simulated	Automatic feature extraction from waveform data	Needs large labeled datasets
[19]	LSTM	Simulated	Time-series fault classification	Training complexity, vanishing gradients
[21]	CNN-LSTM	Simulated	Combined spatial-temporal feature learning	Computationally intensive
[22]	GAN	Simulated	Synthetic fault data generation	Training instability and interpretability issues
[23]	GAN	Simulated	Synthetic fault data generation	Risk of mode collapse, interpretability
**Quantum Machine Learning (QML)**				
[34]	VQC	Simulated (Small)	Circuit-based quantum classifier	Limited qubit scalability
[35]	QML	Simulated (Energy)	Quantum model for load-level disaggregation	Domain-specific, not used for fault detection
[36]	Quantum Classifier	Simulated (Small)	Initial fault classification on small datasets	Not tested in large-scale grids
**Hybrid Quantum-Classical Methods**				
[30]	Quantum-Classical	Image Benchmark	Quantum CNN model for classification tasks	Still in research phase
[31]	Hybrid Model	Simulated (Load Forecasting)	QML with classical pre/post-processing	Not applied to transient fault signals

Despite advancements in fault detection, key research gaps persisted in, including the lack of real-time QML applications (G1), limited use of hybrid quantum-classical models in power systems (G2), no benchmark comparisons with deep learning models (G3), poor model explainability (G4), and reliance on simulated rather than real-world data (G5). The addressing of these gaps is required through innovative integration of QML and classical models for improved real-time fault detection in power systems. Table 2 highlights the research gaps identified in literature.

**Table 2:** Research Gaps Identified in Literature.

Identified Gap Id	Identified Gap	Description	Related Works
**G1**	Lack of QML in real-time fault detection	Few works apply QML to real-time transient classification	[36, 34]
**G2**	Limited hybrid quantum-classical deployment in power systems	Most HQCNN models are focused on finance or vision	[30, 31]
**G3**	No benchmark on hybrid model vs. DL in power faults	No systematic study comparing hybrid and DL on the same grid datasets	[18, 21,30]
**G4**	Explainability in hybrid systems	No research yet on explainable HQCNNs for critical grid applications	[37, 30]
**G5**	Simulated vs. real data generalization	Most work tested only on simulation data; real-world adaptability not explored	[8,9,12,26,33,38]

A comparison of the performance of different fault detection approaches is presented by Tables 3 and 4. Well in controlled environments, conventional methods, including neural networks and wavelet-based techniques, are performed, but noise and complex fault conditions encountered in real-world systems are often failed to be handled. Higher accuracy for both temporal and spatial data is achieved by more advanced models, such as CNNs and LSTMs; however, increased computational demand is incurred by this improvement. Promising accuracy (up to 95%) is shown by quantum-based methods like the Variational Quantum Classifier (VQC), but limitations in scalability and dataset size are faced. Tables 3–5 provide a structured comparison of fault detection approaches, dataset characteristics, and methodological capabilities. A summary of quantitative performance metrics reported in prior studies and in the proposed method is presented in Table 3. A qualitative comparison of classical, deep learning, and hybrid quantum–classical approaches based on reported capabilities in the literature is provided in Table 4. The datasets used in this work, including their source, fault types, and overlap status, are described in Table 5.

**Table 3:** Evaluation Metrics and Performance Comparison of Fault Detection Methods.

Ref. No.	Methodology	Evaluation Metric	Performance Score	Comments
[12]	Neural Networks	Accuracy	90%	High accuracy but struggles with noise handling
[14]	Wavelet Transform	Signal-to-Noise Ratio (SNR)	92%	Effective in clean datasets, less robust in noisy conditions
[9]	SVM	Precision, Recall	85% (Precision), 80% (Recall)	Excellent for binary classification, but struggles with complex faults
[18]	CNN	Accuracy, F1-Score	95%, 93%	Excellent at feature learning but requires large labeled datasets
[19]	LSTM	F1-Score, Loss	90%, 0.1	Strong temporal dependency modeling but resource-intensive
[23]	GAN	FID (Fréchet Inception Distance), Accuracy	0.2 (FID), 85%	Good at generating synthetic data but not real-time
[34]	VQC	Qubit Fidelity	95%	Performs well with small datasets but struggles with larger ones
[30]	QCNN	Accuracy, Circuit Depth	88%, 10^3	Good accuracy, but requires high computational resources
[12]	Neural Networks	Accuracy	90%	High accuracy but struggles with noise handling

**Table 4:** Comparison of Classical, Deep Learning, and Quantum-Based Fault Detection Methods.

Criteria	Classical Techniques	Deep Learning	Quantum & Hybrid Models
Accuracy	Moderate	High	Promising (depends on quantum backend)
Real-Time Capability	High	Medium (depends on model size)	Limited (due to quantum hardware)
Scalability	Good	High (but needs more data)	Low (NISQ limitation)
Interpretability	High	Low (black-box)	Very low
Dataset Requirement	Small	Large	Medium (can work with less due to QML)
Computational Cost	Low	High	High (especially with hybrid simulation)
Suitability for Edge Devices	Yes	Partially	Not yet feasible

**High:** consistently demonstrated across multiple benchmark studies; **Moderate:** demonstrated under task-specific or constrained settings; **Low:** limited empirical evidence or high dependency on tuning.

**Table 5:** Dataset Characteristics for Fault Detection Methods.

Ref. No.	Dataset Type	Dataset Size	Dataset Source	Fault Types	Method	Overlap
[12]	Simulated	10,000 instances	Custom MATLAB/Simulink simulation	Transient, Short-circuit	Neural Networks	No
[14]	Real Fault Logs	1,500 logs	Power Grid Operator	Line faults, Equipment failure	Wavelet Transform	No
[9]	Simulated + Real	5,000 instances	Grid Simulator, Public dataset	Line faults, Overload	Support Vector Machine	No
[18]	Simulated	20,000 samples	Custom fault simulator	Short-circuit, Grounding	CNN	No
[19]	Simulated	15,000 samples	Synthetic fault simulator	Transient faults, Oscillations	LSTM	No
[21]	Simulated	25,000 samples	Power system grid simulator	Transients, Equipment Failure	CNN-LSTM Hybrid	No
**This work**	**Simulated + Real**	**~800 events + simulated cases**	**IEEE 14/39-bus, PMU/SCADA (EPRI, LARIAT)**	**SLG, DL, TP, HIF**	**HQCNN**	**No**

Table 5 is emphasized by how both the type and size of datasets influence the evaluation of fault detection models. Simulated data is relied upon by many studies, which allows a wide range of fault patterns to be learned by models such as neural networks and CNNs. In contrast, smaller real-world datasets are used by some approaches, including wavelet-based methods and support vector machines, leading to more reliable and realistic performance assessments. Very limited sample sizes especially constrain quantum models, such as variational quantum classifiers. Overall, the major challenge in accurately evaluating model robustness and generalization capability is posed by the scarcity of real-world data.

In Table 5, the term overlap is referred to by the authors as relating to whether the same fault events or data samples have appeared across different studies or dataset partitions. In addition, the performance of fault detection methods is compared across various fault categories in Table 6. Transient faults are well identified by neural networks and wavelet-based approaches, while strong results for short-circuit fault detection are shown by CNNs. Ground faults are particularly well modeled by LSTMs because of their ability to capture temporal patterns, and robust performance is demonstrated by hybrid CNN–LSTM models when mixed fault conditions are dealt with. Moderate performance across fault types is shown by quantum-based models, such as Quantum CNNs, but hardware limitations and limited dataset availability currently constrain their effectiveness.

**Table 6:** Fault Detection Performance by Fault Type.

Ref. No.	Fault Type	Methodology	Detection Performance	Key Findings
[12]	Transient Faults	Neural Networks	High	Effective in detecting fault inception but limited by noise
[14]	Short-Circuit	Wavelet Transform	Moderate	Good at handling sharp transitions but weak in multi-fault scenarios
[9]	Line Faults	SVM	High	Performs well for single fault types; struggles with multi-faults
[18]	Short-Circuit	CNN	Very High	Excellent for detecting fault patterns in signal images
[19]	Ground Faults	LSTM	High	Strong in capturing temporal dynamics of faults over time
[21]	Mixed Faults	CNN-LSTM Hybrid	Very High	Combines spatial and temporal features for more complex fault scenarios
[23]	Any Faults	GAN	Moderate	Limited in real-time, good for generating synthetic training data
[30]	Line & Transient Faults	Quantum CNN	Moderate	Performs well on simple faults but struggles with real-world complexity

## 3. Methodology

**Fig 1:**
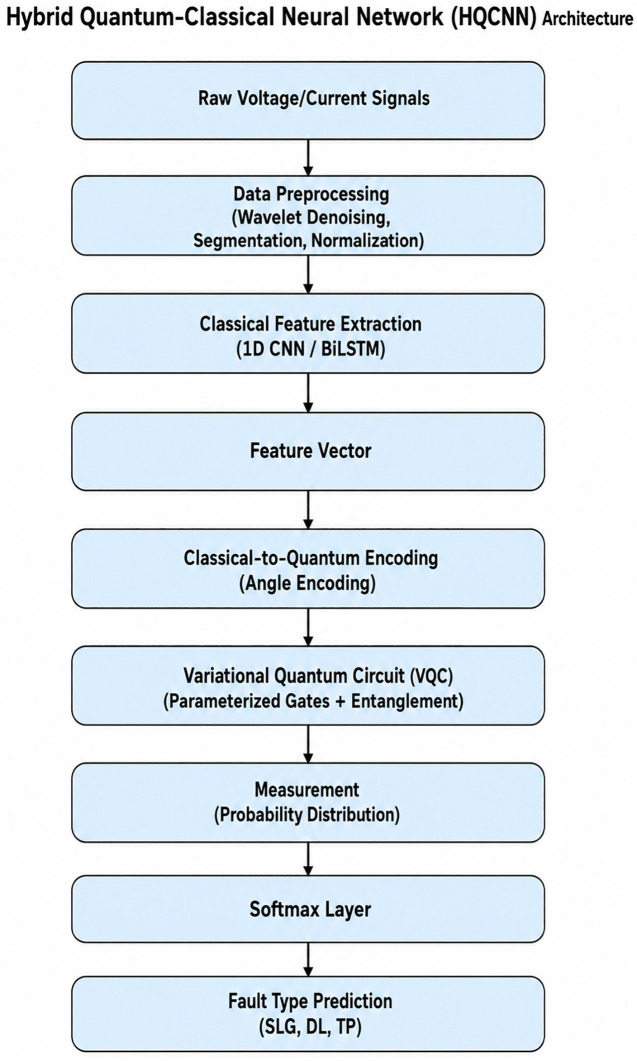
An Improved HQCNN for Real-Time Fault Detection.

**Fig 2:**
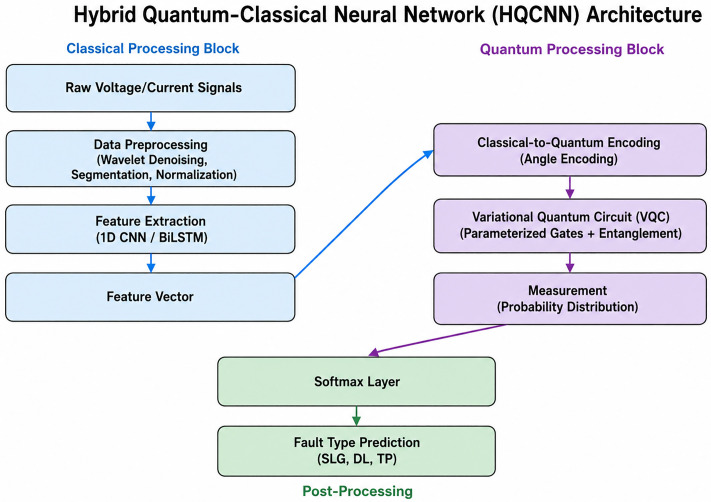
Enhanced Version of HQCNN Architecture for Real-Time Fault Detection.

**Fig 3:**
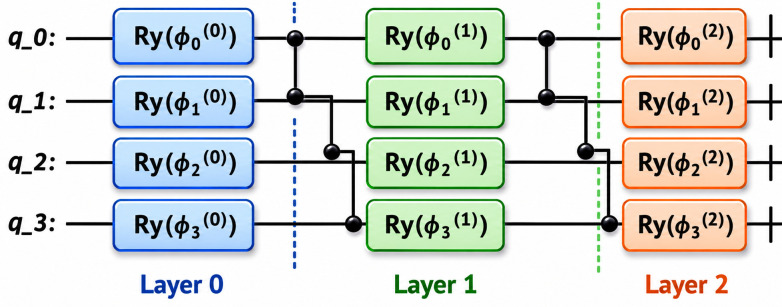
Four-qubit, three-layer variational quantum circuit with parameterized rotations and linear CZ entanglement.

The suggested framework for real-time fault detection in power systems is utilized by an HQCNN (Hybrid Quantum-Classical Neural Network), which integrates with deep learning for extracting the features and a quantum variational circuit for classification, as illustrated in Figs 1–3. The shortcomings of entirely classical models regarding both accuracy and inference speed are aimed to be overcome by this architecture, especially in the identification of transient faults within high-resolution time-series data.

### 3.1. Framework architecture

The HQCNN architecture consists of three major components: (i) a classical feature extraction module using either a 1D CNNor a BiLSTM network, (ii) a classical-to-quantum encoding layer, and (iii) a Variational Quantum Classifier (VQC).

Given a raw signal $X\in R^{n\times t}$, where, n is used as the count of sensors and t is the term used to show the length of the time window, the objective is to predict fault classes y∈ {0,1,...,k} through a non-linear mapping composed of classical and quantum transformations. The overall formulation is given as:


y=(argmaxσjj)(VQC(ϕ(F(X)))) (1)

where F(X) is the classical feature extraction function, ϕ denotes the quantum encoding, and VQC refers to the quantum variational circuit. The function σ represents the softmax operator that transforms the quantum measurement probabilities into a probabilistic class prediction.

The input signal X, representing a segment of voltage or current waveforms from multiple sensors, is initially processed by a classical feature extraction function F(X), implemented using either a 1D Convolutional Neural Network (CNN) or a Bidirectional Long Short-Term Memory (BiLSTM) network. The extracted features are mapped to a quantum state through an encoding function ϕ(·) and processed by a variational quantum circuit (VQC).The final output y^ is calculated through a Softmax activation as:


$$\begin{array}{c} \hat{y}=\sigma \;\left(\text{VQC}\;\left(\phi \;\left(F(X)\right)\right)\right) \end{array}$$ (2)

### 3.2. Data collection and preprocessing

For testing the suggested model, simulated and actual data were utilized. Simulated datasets have been created based on the IEEE 14-bus test system through MATLAB/Simulink under different fault scenarios such as single-line-to-ground (SLG), double-line (DL), and three-phase (TP) faults. Actual data was obtained from PMU/SCADA logs of open-access sources such as EPRI and LARIAT. The training and testing dataset includes both simulated and actual data. Simulated fault cases were created based on the IEEE 14-bus and 39-bus test systems, including single-line-to-ground (SLG), double-line (DL), and three-phase (TP) faults with different fault resistances and inception angles. The dataset in the real world includes about 800 events gathered from Phasor Measurement Units (PMUs) and SCADA logs, retrieved from open-source sites like EPRI and LARIAT. These field occurrences comprise events of single-line-to-ground, double-line, three-phase, and high impedance faults (HIF). Simulated data were neat and well-synchronized at 50/60 Hz sampling, whereas field data needed extra steps of preprocessing.The simulated datasets and real-world PMU/SCADA datasets are non-overlapping; training and controlled evaluation were conducted using simulated data, while validation and testing were carried out using real-world events only. All real-world PMU/SCADA data used in this study were obtained from publicly accessible repositories associated with smart grid monitoring projects, and no restricted or proprietary datasets were accessed. Wavelet-based denoising was done more aggressively to counteract ambient noise, event synchronization was also fixed by cross-correlation methods, and missing data in short bursts (less than 20 ms) were filled linearly for maintaining temporal coherence.

Following preprocessing, fixed length overlapping windows were created from the synchronized voltage and current signals to capture localized fault dynamics. To prevent information leakage, data splitting was conducted at the fault-event level, ensuring that all windows derived from a given event were assigned exclusively to either the training, validation, or test set.

### 3.3. Data preprocessing and feature normalization

As signal quality is important aspect, for this, several important steps taken here for data preprocessing to ensure consistent signal quality. Initially, wavelet-based denoising is applied to detect and remove high-frequency components that were not related to fault events. Then the signals were divided into fixed 5-cycle segments to keep the input size balanced and uniform. At the end, Min–Max normalization was used to scale down all features to the range of 0–1, to make the data suitable for quantum encoding.

During preprocessing, the x is used as raw signal and it is cleaned from noise by applying Discrete Wavelet Transform, in which it is broken down into approximation coefficients named Aj and detail coefficients named Dj in eq. 3.


$$\begin{array}{c} x=Aj+Dj \end{array}$$ (3)

To enhance the models ability to generalize across different grid scenarios, preprocessing was applied. First, the signal is denoised using Discrete Wavelet Transform (𝐷𝑊𝑇), where a signal (𝑡) is decomposed into approximation 𝐴𝑗(𝑡) and detail coefficients 𝐷𝑗(𝑡).


$$\begin{array}{c} x(t)=Aj(t)+\sum_{i=\text{1}}^{j}Di(t) \end{array}$$ (4)

Denoising is performed by thresholding Di(t) and reconstructing the signal via Inverse DWT(IDWT). After denoising, signals are normalized between [0,1] using Min-Max normalization.


$$\begin{array}{c} x_{\text{norm}}=\frac{x-\underset{\text{train}}{\text{min}}}{\underset{\text{train}}{\text{max}}-\underset{\text{train}}{\text{min}}} \end{array}$$ (5)

To avoid information leakage and preserve event-level amplitude characteristics that are critical for fault discrimination, Min–Max normalization statistics is computed once on training dataset independently. It is done for each signal channel. The same normalization parameters are then applied unchanged to the validation and test datasets. Here, Normalization is not performed on a per-window basis. The data is then segmented into fixed-size windows of 5 cycles (100 ms for 50 Hz systems), with each segment forming an input sample.

Dataset Composition and Split: The complete dataset consists of both simulated and real-world fault events. Simulated data were generated using the IEEE 14-bus and 39-bus test systems, while approximately 800 real-world fault events were collected from PMU/SCADA repositories. The dataset includes balanced representations of SLG (singlelinetoground), DL (double-line), and TP (three-phase) faults to avoid class bias. The combined dataset was divided into 70%, 15%, and 15% for training, validation and testing, respectively, at the fault-event level to prevent information leakage. The same data split was used to generate the results reported in Fig 6 and Table 7.

**Table 7:** Comparative performance of models on simulated IEEE 14-bus data.

Model	Accuracy (%)	Precision (%)	Recall (%)	F1-score (%)	Inference Latency (ms)
**CNN**	93.12	91.87	92.45	92.15	2.1
**BiLSTM**	94.06	93.15	92.79	92.97	3.6
**QVC**	88.74	87.42	86.91	87.16	1.8
**HQCNN**	96.43	95.92	96.08	96.00	2.7

**Fig 4:**
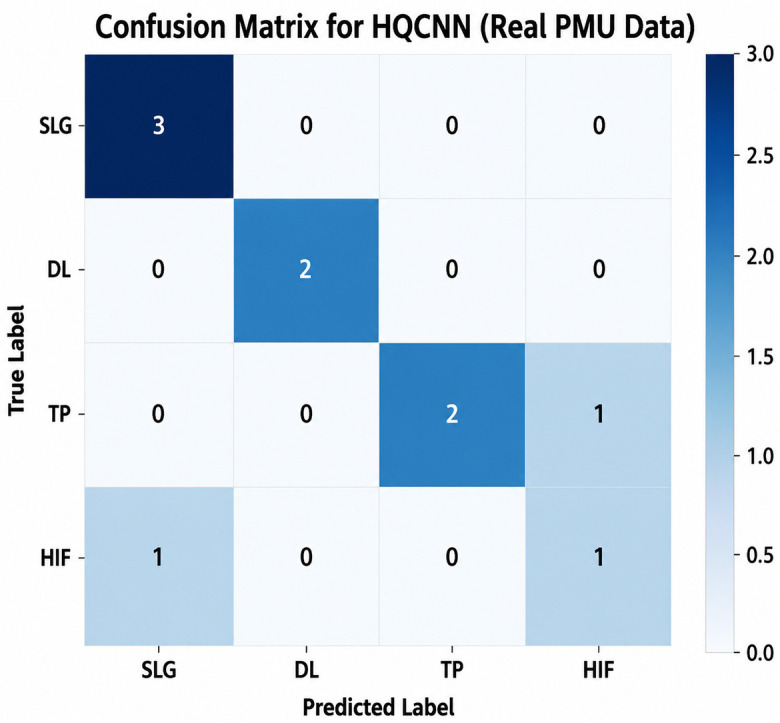
Confusion Matrix.

**Fig 5:**
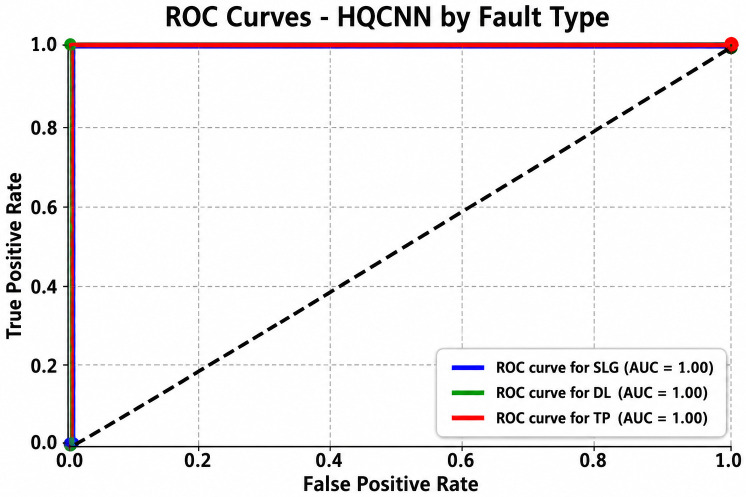
ROC Curves- HQCNN by Fault Type. The x-axis is showing the predicted fault classes, whereas the y-axis is showing the true fault labels.

### 3.4. Classical feature extraction

The traditional component of the HQCNN is responsible for extracting high-level features from the pre-processed input. Two network architectures were considered as alternatives. The first one employs a 1D CNN consisting of convolutional layers with ReLU activations, max pooling, and fully connected layers. This configuration effectively extracts spatial correlations in waveform patterns. The second setup utilizes a BiLSTM to identify temporal dependencies that are of particular importance when detecting the propagation and evolution of faults over time.

The feature extractor F(X) is either a 1D CNN or a BiLSTM network. For CNN, the output of a convolutional layer is given as:


$$\begin{array}{c} \overset{\left(l\right)}{\underset{i}{h}}=\sigma (\sum_{k=\text{1}}^{K}\overset{\left(l\right)}{\underset{k}{w}}*xi-k+b^{\left(l\right)}) \end{array}$$ (6)

where $\overset{\left(l\right)}{\underset{k}{w}}$ is the filter weight at layer l, $b^{\left(l\right)}$ is the bias, and σ(·) is a non-linear activation such as ReLU. In BiLSTM, the hidden state at time t is updated by combining forward $\overset{\to }{h_{t}}$ and $\overset{\leftarrow }{h_{t}}$backward states:


$$\begin{array}{c} ht=[\overset{\leftarrow }{h_{t}};\overset{\to }{h_{t}}] \end{array}$$ (7)

The final output vector $z\in R^{d}$ encapsulates temporal-spatial information and is passed to the quantum encoder.

### 3.5. Classical-to-quantum encoding

For quantum encoding, each classical feature zi is mapped to a qubit’s rotation angle using the Ry gate:


$$\begin{array}{c} Ry(zi)=e^{-iziY/2} \end{array}$$

The encoded quantum state is the tensor product across all qubits. For enabling quantum processing, the classical vector w is encoded into a quantum state through angle encoding (also known as parameterized rotation encoding). Every scalar zi is translated into the angle for rotation of a quantum gate. The classical output w=[w1,w2,…,wV] is encoded through quantum states using angle encoding:


$$\begin{array}{c} |\psi i\rangle =RY(\theta i)|0\rangle ,where\theta i=\pi (2z_{i}-1) \end{array}$$ (8)

This centred angle encoding preserves sign information and improves the expressive capacity of shallow variational quantum circuits while remaining compatible with NISQ hardware constraints.

The complete quantum state is:


$$\begin{array}{c} |\psi \rangle =\overset{d}{\underset{i=1}{⨂}}RY(\pi \cdot zi)|0\rangle \end{array}$$ (9)

This process yields a multiqubit entangled input state for the variational quantum circuit. The quantum variational circuit computes the expectation value of Pauli-Z operators on every qubit:


$$\begin{array}{c} \langle Zi\rangle =\langle \psi |Zi|\psi \rangle \end{array}$$ (10)

The resulting expectation values are passed through the Softmax function to achieve the various class probabilities:


$$\begin{array}{c} y=Softmax\left(\left\langle Z\right\rangle \right) \end{array}$$ (11)

The network is trained by the cross-entropy loss for minimization:


L=−∑iyilog(y^i) (12)

where$y_{i}$ is used to represent the true labels, y^i is showing the predicted class probabilities. A hybrid optimization procedure jointly updates classical parameters θc and quantum parameters θq by minimizing:


minL(yi,y^i)θcθq+λ∣∣θq∣∣2 (13)

where λ controls the regularization strength on quantum parameters.This unified loss function is used throughout the hybrid training process to jointly optimize classical and quantum parameters.

### 3.6. Variational quantum classifier (VQC)

The quantum classification phase employs a parameterized quantum circuit to be run on the encoded input. The quantum variational layer employed in this work runs on four qubits, providing adequate expressiveness with compatibility for existing NISQ hardware. The quantum circuit depth is fixed at three layers, where every layer consists of parameterized single-qubit rotations and entangling operations. Entanglement is introduced through Controlled-Z (CZ) gates between neighbouring qubits following each rotation block. Controlled-Z (CZ) gates were chosen for entanglement as they preserve the computational basis structure and are natively supported on several quantum hardware platforms, while offering equivalent entangling capability to CNOT gates for variational circuits. This entanglement is used to preserve non-linear correlations between features in an efficient manner. To reduce the effect of noise byhardware, simple measurement error mitigation procedures were utilized using Qiskit Ignis calibration routines. Only measurement (readout) error mitigation using Qiskit Ignis calibration routines was applied; no full quantum error correction codes were implemented.

Furthermore, the Simultaneous Perturbation Stochastic Approximation (SPSA) optimizer was chosen to train the quantum parameters, due to its stability in noisy environments and effectiveness in approximating gradients from sparse quantum measurements. Each variational layer consists of trainable singlequbit rotation gates $R_{y}(\overset{\left(l\right)}{\underset{j}{\phi }})$ applied to all qubits, followed by Controlled-Z (CZ) gates between adjacent qubits to introduce entanglement. The rotation angles $\overset{\left(l\right)}{\underset{j}{\phi }}$are optimized during training using the SPSA optimizer as part of the hybrid quantum–classical learning process.

The quantum circuit consists of repeating layers of singlequbit parameterized rotation gates and entanglement gates (e.g., CZ or CNOT). The variational circuit output is a probability distribution over measurement outcomes ∣y⟩, for which expected value for a Pauli-Z observable on qubit i is:


$$\begin{array}{c} \langle Z_{i}\rangle =\langle \psi (\theta )|Z_{i}|\psi (\theta )\rangle \end{array}$$ (14)

The expectation values obtained from the variational quantum circuit are passed through a softmax function to produce class probabilities. Model training is performed by minimizing the unified cross-entropy objective defined in Equation (13), with quantum parameters optimized using the SPSA algorithm.

### 3.7. Hybrid training and optimization strategy

The HQCNN model got trained end-to-end by a hybrid optimization approach. Classical parameters (CNN/BiLSTM weights) are updated using stochastic gradient descent, while quantum parameters are optimized on simulators or real quantum hardware via the Simultaneous Perturbation Stochastic Approximation (SPSA) algorithm. Let 𝐰denote the classical parameters and θthe quantum parameters. Training is performed by minimizing the unified objective function defined in Equation (13), which jointly optimizes both classical and quantum components with a regularization term controlling the contribution of the quantum parameters.

### 3.8. Evaluation setupand evaluation metrics

The proposed HQCNN model was developed using PyTorch for the classical deep learning components and Qiskit Machine Learning for implementing the variational quantum circuit. Experiments were carried out on a high-performance computing system equipped with an Intel Xeon processor, an NVIDIA RTX 4090 GPU, and 64 GB of memory, which supported efficient execution of both classical and quantum operations.

The variational quantum circuit is built from repeated layers of parameterized single-qubit rotation gates and Controlled-Z (CZ) entangling gates, as outlined in Section 3.5. Expectation values of Pauli-Z operators are computed using measurement outcomes from the circuit, which serve as inputs for the fault classification task. Common classification metrics, including accuracy, precision, recall, and F1-score, are used to assess model performance, derived from true positive, true negative, false positive, and false negative results. Inference time is also measured to evaluate real-time applicability. All results are reported for each fault category and averaged using five-fold cross-validation to improve reliability and reduce variability.

## 4. Experimental results and analysis

In this section the performance is analysedfor the HQCNN (Hybrid Quantum-Classical Neural Network) is presented using simulated and real-world datasets. Here, the aim is related to the evaluation of its performance in detecting faults accurately under different operating conditions and to compare it with current classical approaches.

### 4.1. Performance on simulated data

The performance of the HQCNN against the common CNN, BiLSTM, and shallow quantum-only classifier (QVC) is collated in Table 7. Its counterparts were consistently outperformed by the HQCNN across all metrics.

**Fig 6:**
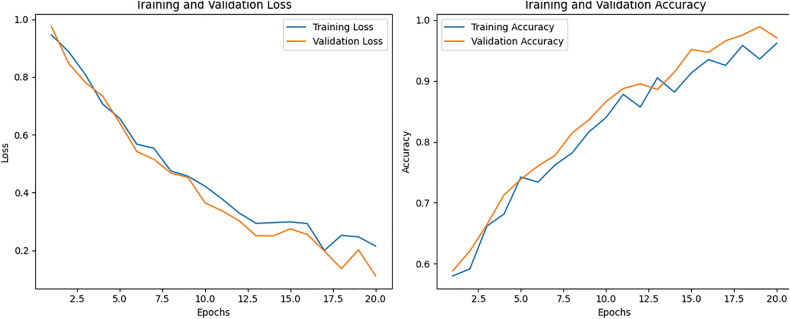
Training and validation loss (at the left) and accuracy curves (at the right) for the proposed HQCNN model‌‌.

As Table 7 is showing the best accuracy (96.43%) and F1-score (96.00%) were achieved by the HQCNN, while a competitive inference latency of 2.7 ms was maintained. The observed performance gain is characterized to the ability of the quantum variational layer to model complex non-linear feature interactions through parameterized rotations and entangling operations, which can enhance decision boundaries in ambiguous fault scenarios. Fig 4 is showing Confusion Matrix of HQCNN on Real PMU Data and Fig 5 is showing ROC Curves. Training and Accuracy loss is showing by the Fig 6.

### 4.2. Fault-type detection accuracy

To further understand model behaviour, the detection accuracy was broken down by fault type. Table 8 presents fault-wise classification results.

**Table 8:** HQCNN performance by fault type (simulated dataset).

Fault Type	Accuracy (%)	Precision (%)	Recall (%)	F1-score (%)
Single-Line-Ground (SLG)	97.32	96.88	97.15	97.01
Double-Line (DL)	95.64	94.97	95.23	95.10
Three-Phase (TP)	96.71	96.32	96.55	96.43

The HQCNN model demonstrated robust performance across all fault types, with slightly lower scores for double-line faults, possibly due to their similarity in signal characteristics with SLG events in some topologies.

### 4.3. Generalization on real-world data

To evaluate real-world applicability, the model was tested on PMU data collected from an open-access smart grid monitoring project. Table 9 shows the results.

**Table 9:** HQCNN generalization performance on real PMU dataset.

Model	Accuracy (%)	F1-score (%)	Inference Latency (ms)
CNN	90.21	89.45	2.4
BiLSTM	91.38	90.91	4.1
QVC	86.09	85.37	2.0
**HQCNN**	**94.74**	**94.19**	**2.9**

**Fig 7:**
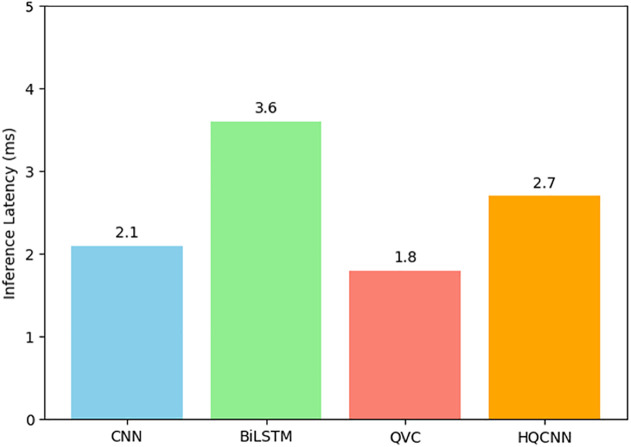
Inference Latency Comparison Across Models.

Even under noisy and asynchronous conditions in real-world settings, the HQCNN retained high predictive capability and low inference latency, demonstrating its suitability for deployment in real-time energy management systems. Fig 7 depicts the Inference Latency Comparison Across Models.

### 4.4. Ablation study

To assess the individual contribution of the quantum layer, an ablation study was conducted. Results are presented in Table 10.In this ablation study, the quantum variational layer was removed and replaced with a fully classical dense layer of comparable size, while keeping all other network components and training settings unchanged. No hyperparameters were tuned during the ablation study; this design isolates the performance impact of the quantum layer itself.

**Table 10:** Impact of quantum component on classification performance.

Configuration	Accuracy (%)	F1-score (%)
Classical Only (CNN + FC)	93.12	92.15
Classical + Quantum (HQCNN)	**96.43**	**96.00**

The 3.31% improvement in accuracy confirms that the variational quantum layer adds substantial discriminative power to the overall network.

## 5. Conclusion and future work

An HQCNN architecture for real-time fault detection in power transmission systems was introduced in this paper. A 1D CNN or BiLSTM-based classical feature extractor was integrated with a four-qubit parameterized quantum circuit (PQC) for classification, with angle encoding and entanglement leveraged via Controlled-Z gates. Complete training of the model was conducted using a hybrid optimization strategy, which combined stochastic gradient descent for classical parameters and the SPSA (Simultaneous Perturbation Stochastic Approximation) algorithm for quantum parameters. Comprehensive experiments were conducted on both simulated datasets generated from IEEE 14-bus and 39-bus systems and real-world Phasor Measurement Unit (PMU) datasets containing approximately 800 real fault events. Fault types evaluated included SLG, DL, TP, and HIF.An overall accuracy 96.43% on simulated data and 94.74% on real-world PMU data was achieved by the HQCNN, with conventional CNN and BiLSTM baselines being outperformed. Inference latency was maintained within 2.7 milliseconds on simulated environments and under 3 milliseconds on real PMU data, with potential for real-time deployment demonstrated. It was confirmed by ablation studies that classification performance was improved by 3.31% over purely classical networks through the inclusion of the quantum variational layer.

Despite the encouraging performance, several challenges remain for practical deployment. Computational resources on edge devices were limited by real-time latency constraints, and widespread adoption continues to be restricted by noise in NISQ (Noisy Intermediate-Scale Quantum) hardware. In addition, although strong robustness was demonstrated by HQCNN, questions related to model interpretability and transparency in fault decision-making remain unresolved. These limitations can be addressed in several ways by future research. First, the proposed HQCNN should be evaluated using larger and more diverse real-world PMU datasets to better assess its generalization across different grid configurations and operating conditions. Second, transparency would be enhanced, operator confidence would be improved, and regulatory requirements would be supported by incorporating explainable AI techniques tailored to hybrid quantum–classical models.Finally, versions of the HQCNN that are simplified and resource-efficient should be designed for deployment on edge devices in substations, ensuring an effective balance between detection accuracy and computational cost.

Finally, the feasibility of HQCNNs as quantum technology matures will be improved through the exploration of more advanced quantum noise mitigation methods and scalable circuit ansatzes.The proposed HQCNN framework is designed and evaluated for single-fault classification scenarios, where one dominant fault type is present at a given time. Simultaneous multi-fault detection was not considered in this study. The framework could be extended to multi-label fault detection by modifying the output layer and loss formulation, which is a potential direction for future work.
