# Supplementary material for: Hybrid quantum-classical neural networks for real-time fault detection in power systems
Source: PLoS One. 2026 Jun 18;21(6):e0349887. doi: 10.1371/journal.pone.0349887 (PMC13278437; doi:10.1371/journal.pone.0349887)
Supplement: Attachment — Submitted filename: Manuscript_Review_Normalisation_VQC.docx [file pone.0349887.s001.docx]

**I have reviewed the manuscript and it presents a great use case for fault detection in power systems . The methods are simple and replicable and is practical in NISQ devices. How ever I have some minor concerns related to encoding , VQC and Use of optimizers .**

**1) Normalisation scope is unspecified (window-wise vs train-set)**

**Manuscript text:**

“Finally, Min–Max normalization scales the data between [0,1], preparing consistent inputs for the feature extraction stage.”

“After denoising, signals are normalized between [0,1] using Min–Max normalization: x_norm = (x − min(x)) / (max(x) − min(x)).”

**My concern:**

The scope of min/max is not stated. If computed per window, this can erase event-level amplitude cues in PMU data. Please specify that statistics are computed on the training set per channel to avoid leakage and amplitude suppression.

**Suggested fix:**

• State explicitly: normalisation statistics are computed once on the training set per channel; apply the same mapping to validation/test.

• Avoid per-window Min–Max; it distorts cross-event amplitude comparisons.

**Relevant equations (LaTeX):**

*$$ x_{\text{norm}}=\dfrac{x-\min_{\text{train}}}{\max_{\text{train}}-\min_{\text{train}}} $$*

**2) Angle encoding restricts rotations to [0, \pi]**

**Manuscript text:**

“For quantum encoding, each element z_i is mapped to a rotation state via |ψ_i⟩ = R_y(θ_i)|0⟩, where θ_i = π · z_i.”

**My concern:**

Because z_i ∈ [0,1], θ_i ∈ [0,π]. This folds sign information and compresses dynamic range, reducing expressivity of a shallow VQC.

**Suggested fix:**

• Use a centered mapping to preserve sign: θ = π(2z − 1) so θ ∈ [−π, π].

• Alternatively, z-score on train set then squash: θ = π·tanh((x−μ)/σ).

• If you re-upload data across layers, note it—it mitigates shallow depth limits.

**Relevant equations (LaTeX):**

*$$ \theta_i=\pi (2 z_i - 1) \in [-\pi,\pi] $$*

*$$ \theta_i=\pi\,\tanh\!\left(\dfrac{x_i-\mu_i}{\sigma_i}\right) $$*

**3) Figure shows fixed Ry(0.5) while text claims parameterized rotations**

**Manuscript text:**

“Figure 3 depicts a 4-qubit, 3-layer circuit where each layer applies Ry(0.5) to all qubits, followed by CZ entanglers.”

“The quantum circuit depth is fixed at three layers, where each layer consists of parameterized single-qubit rotations and entangling operations.”

**My concern:**

This is contradictory: is 0.5 a fixed bias, or are the angles trainable? Readers will assume either a constant offset or a true variational parameter set.

**Suggested fix:**

• If 0.5 was illustrative, change the figure to Ry(ϕ_j) and say ϕ_j are trainable.

• If a fixed bias is intentional, justify it (stability) and provide a micro-ablation over {0, 0.25, 0.5, 1.0} rad.

**Relevant equations (LaTeX):**

*$$ |\psi\rangle=\prod_{\ell=1}^{L}\Big(\text{CZ}\cdot \prod_{j=1}^{n} R_y(\phi_{j}^{(\ell)})\Big) |0\rangle^{\otimes n} $$*

**4) Objective/optimiser story: SPSA stated; simulated annealing not documented**

**Manuscript text:**

“The expectation values are passed through Softmax and trained via cross-entropy loss.”

“SPSA optimizer was chosen to train the quantum parameters due to robustness to noise.”

**My concern:**

If simulated annealing was used (init or depth search), it is not documented (no schedule or role). If not used, remove stray mentions to avoid confusion.

**Suggested fix:**

• Add SPSA hyper-parameters (a, c, decay), perturbations/step, shots/update, and seeds.

• If annealing is used for initialisation: include schedule (T0, decay, steps) and clearly state the handoff to SPSA.

**Relevant equations (LaTeX):**

*$$ \min_{\theta_c,\theta_q}\; \mathcal{L}_{\text{CE}}(y,\hat y) + \lambda \lVert \theta_q \rVert_2^2 $$*

*$$ \hat y = \mathrm{Softmax}(W\,\langle Z \rangle + b) $$*

**5) Two hybrid-objective notations—align or define equivalence**

**Manuscript text:**

“A hybrid optimization jointly updates classical θ_c and quantum θ_q via L(y,ŷ) + λ||θ_q||^2.”

“The joint objective is L_total = L_CE + λ · L_VQC.”

**My concern:**

These read as two different regularisations unless L_VQC = ||θ_q||^2 is stated. Align the notation or define equivalence.

**Suggested fix:**

• Define L_VQC explicitly as an L2 penalty on quantum parameters, or use one canonical form everywhere.

**Relevant equations (LaTeX):**

*$$ \mathcal{L}_{\text{total}} = \mathcal{L}_{\text{CE}} + \lambda \lVert \theta_q \rVert_2^2 $$*

*$$ \text{or}\quad \mathcal{L}_{\text{total}} = \mathcal{L}_{\text{CE}} + \lambda\,\mathcal{L}_{\text{VQC}},\;\; \mathcal{L}_{\text{VQC}} \equiv \lVert \theta_q \rVert_2^2 $$*

**6) Measurement-error mitigation is referenced but unspecified**

**Manuscript text:**

“Simple measurement error mitigation procedures were utilized using Qiskit Ignis calibration routines.”

**My concern:**

Specify which routine (local readout mitigation via calibration matrices?) and the re-calibration cadence; include a small with/without table on a held-out slice.

**Suggested fix:**

• Name the routine (e.g., LocalReadoutMitigator) and report calibration frequency (e.g., every N batches).

• Report accuracy with and without mitigation to show its effect.

**7) Guard against split leakage across windows from the same event**

**Manuscript text:**

“Signals are segmented into fixed-length windows and normalized to a common range before classification.”

**My concern:**

Please confirm that windows from the same PMU event do not straddle train/test splits; otherwise, correlation can inflate reported accuracy.

**Suggested fix:**

• Add a one-line note: events are split at the event level; windows from any single event reside in exactly one split.

**8) Unify Softmax/VQC output notation**

**Manuscript text:**

“ŷ = Softmax(Q(E(F(x))))”

“y = Softmax(⟨Z⟩)”

**My concern:**

These are conceptually aligned but differ notationally. State that ⟨Z⟩ denotes the VQC readout vector and use one canonical form.

**Suggested fix:**

• Use ŷ = Softmax(VQC(E(F(x)))) throughout, with ⟨Z⟩ explicitly defined as the VQC expectation vector.

**Relevant equations (LaTeX):**

*$$ \hat y = \mathrm{Softmax}\!\big(\mathrm{VQC}(E(F(x)))\big) $$*

*$$ \langle Z \rangle \equiv \big(\langle Z_1\rangle,\dots,\langle Z_K\rangle\big) $$*

**What I liked :**

• Strong application fit (real-time PMU fault detection).

• Pragmatic NISQ-friendly VQC (few qubits, shallow depth, CZ entanglers).

• Clear hybrid training loop (cross-entropy, SPSA).

• Solid preprocessing chain; minor scope clarification will make it airtight.

• Dual data regime (simulation + field events) enhances external validity.
