# Supplementary material for: Hybrid quantum-classical neural networks for real-time fault detection in power systems
Source: PLoS One. 2026 Jun 18;21(6):e0349887. doi: 10.1371/journal.pone.0349887 (PMC13278437; doi:10.1371/journal.pone.0349887)
Supplement: Attachment — Submitted filename: PONE-D-25-43455.docx [file pone.0349887.s002.docx]

Shree ganeshaya namaha

Paper Id: **PONE-D-25-43455**

**Title:** Hybrid Quantum-Classical Neural Networks for Real-Time Fault Detection in Power Systems

1. The purpose of Table 3, 4, and 5 and their corresponding columns is not very clear.
2. In Table 4, the meaning or measure of the various entries under the column 'classical techniques' and 'deep learning' is not very clear. What does moderate, high and other keywords stand for and how is this measured?
3. In Table 5 lists the various datasets, are these dataset overlapping? Their names if any, needs to be specified.
4. Font size of Fig1, Fig2 needs to be increased as they are not readable
5. The Figure 3 can be accommodated as a whole.
6. The representation of the variables in different equation should be consistent for a given variable. Eqn 1 and eqn 2 has different representation for the input signal.
7. In Fig5, the role of ‘even synchronization’ and ‘missing data handling’ is not clear. The paper begins with taking a signal and cutting it into parts. Request you to justify the missing data.
8. In VQC, why have you used CZ gates for entanglement and why not CNOT gate? Justification for the same is needed.
9. If you are using abbreviations for a particular word then be consistent and use the same throughout the paper. There is no need to use both the full form and abbreviation ahead in the paper.
10. To represent i^th^ instance in an equation use subscript. Eqn 14, 15, and 16 subscripts are not properly represented.
11. I feel Eqn 15 is not correctly printed.
12. Equation 15 and 16 are not a part of the subsection 3.5. Similarly eqn 11, 12 and 13 are not related to subsection 3.4
13. Subsection 3.7 needs to be rewritten as it not very clear.
14. For Fig 6. and Table 7 what is the split ratio? How many samples were used for training and testing? Subsection 3.2 speaks about the data gathering but doesn’t tell us how many samples were simulated and how many of them actually collected and total count in terms of (SLG), double-line (DL), and three-phase (TP) faults. This information might is very much nesseary to see if the dataset is balanced.
15. What does the X and Y labels of confusion matrix stand for?
16. Fig. 8 and 9 shows training and validation accuracies/loss using for which particular techniques?
17. You are using the data from the project 'smart grid monitoring project' are proper permissions sought from the concerned authority?
18. In subsection 4.5, please explain the meaning of the 'An ablation study was conducted to assess the individual contribution of the quantum layer'. What hyper parameter was tuned in this ablation study?
19. This work speaks about applying error correction, what form of error corrections is implemented?
20. Can your proposed techniques of HQCNN detect multiple faults? If Yes, a justification needs to be given in the form of results comparing the various possible combinations.
21. Can you justify this statement used in your paper, 'The performance gain is attributed to the ability of the quantum layer to capture high-dimensional entanglements in the feature space, enhancing decision boundaries for ambiguous cases'.

In summary it would have been appreciated if you could have compared your work with some more state of art techniques. A clear explanation on why a particular gate is contributing or a though study on various entanglement (Linear, Circular and Full) would be appreciated. Is this work considering multiple faults? If yes, a detailed study of the same needs to be added. How is the RY gate contributing to your study? Why quantum or how is quantum helping here needs light.

I personally have the following concerns regarding data:

1. I was not able to verify the availability of any of the dataset used. The paper says they are creating a dataset with some simulated values and certain values taken from the project 'smart grid monitoring project'. Are proper permissions sought from the concerned authority data or citing to the data is missing. The size of the final dataset used in the experiment is missing.
2. The work is technically strong. But still needs a in depth study in terms of entanglement and gate usage. Why quantum is not answered satisfactorily.
3. With the above said revisions the paper can be accepted.
