# Supplementary material for: Hybrid quantum-classical neural networks for real-time fault detection in power systems
Source: PLoS One. 2026 Jun 18;21(6):e0349887. doi: 10.1371/journal.pone.0349887 (PMC13278437; doi:10.1371/journal.pone.0349887)
Supplement: Attachment — Submitted filename: Response_to_Reviewers.docx [file pone.0349887.s004.docx]

**Manuscript ID:** PONE-D-25-43455
**Title:** *Hybrid Quantum-Classical Neural Networks for Real-Time Fault Detection in Power Systems* **Response to Reviewer #1**

| **Reviewer #1 Comment** | **Action Taken / Response** | **Location of Change in Manuscript** | **Reviewer Comment ID (used in manuscript)** |
| --- | --- | --- | --- |
| 1. Clarify whether normalization is performed per window or across the dataset. | Clarified that Min–Max normalization parameters are computed using only the training set and then applied unchanged to validation and test sets to avoid information leakage and preserve amplitude characteristics. | Section 3.3 – Data Preprocessing and Feature Normalization | R1.1 |
| 2. Angle encoding limits expressivity due to restricted rotation range. | Updated the encoding scheme to a centered angle mapping to preserve sign information and enhance expressivity for shallow variational quantum circuits. | Section 3.5 – Classical-to-Quantum Encoding, Equation (8) | R1.2 |
| 3. Quantum circuit figure shows fixed rotation angles instead of trainable parameters. | Redrew the quantum circuit to explicitly depict trainable rotation gates (R_y(\phi_j^{(\ell)})) for all qubits and layers, ensuring consistency between text and figure. | Figure 3, Section 3.6 – Variational Quantum Classifier (VQC) | R1.3 |
| 4. Optimizer choice for quantum parameters is unclear. | Explicitly stated that the SPSA optimizer is used exclusively for training quantum parameters due to its robustness in noisy NISQ environments. | Sections 3.6 and 3.7 | R1.4 |
| 5. Multiple or inconsistent loss formulations appear in the manuscript. | Unified the training objective by defining a single loss formulation and removing redundant loss equations, ensuring consistency across all sections. | Section 3.7 – Hybrid Training and Optimization Strategy | R1.5 |
| 6. Error correction claims may be overstated. | Clarified that only measurement (readout) error mitigation using Qiskit Ignis is applied, and no full quantum error correction codes are implemented. | Section 3.6 – Variational Quantum Classifier (VQC) | R1.6 |
| 7. Possible train–test leakage due to window-based segmentation. | Added an explicit statement confirming that data splitting is performed at the fault-event level so that all windows from a given event belong exclusively to one dataset split. | Section 3.2 – Data Collection and Preprocessing | R1.7 |

**Response to Reviewer #2**

| **Reviewer #2 Comments** | **Action Taken / Response** | **Location of Change in Manuscript** | **Reviewer Comment ID (used in manuscript)** |
| --- | --- | --- | --- |
| 1. Purpose of Tables 3–5 is unclear. | Added an explanatory paragraph describing the intent and scope of Tables 3–5. | Section 2.4 Paragraph immediately preceding Tables 3–5 | R2.1 |
| 2. Qualitative terms in Table 4 (High, Moderate, etc.) are unclear. | Added a footnote defining all qualitative descriptors based on reported empirical evidence. | Section 2.4 Table 4 footnote | R2.2 |
| 3. Dataset overlap in Table 5 is unclear. | Added an explicit “Overlap” column, clarified non-overlapping datasets, and included a row describing the dataset used in this work. | Table 5; Section 3.2 | R2.3 |
| 4. Figures 1 and 2 are not readable. | Regenerated Figures 1 and 2 with increased font size and improved resolution. | Figures 1 and 2 | R2.4 |
| 5. Figure 3 should be presented as a single figure. | Redrew Figure 3 as a unified four-qubit, three-layer variational quantum circuit with corrected labels. | Figure 3 | R2.5 |
| 6. Inconsistent variable notation across equations. | Unified notation across all equations using consistent symbols for inputs, encoding, and quantum operations. | Sections 3.1–3.5 | R2.6 |
| 7. Role of missing data handling is unclear. | Expanded preprocessing description to include synchronization, denoising, and linear interpolation for short missing segments. | Section 3.2 | R2.7 |
| 8. Justification for using CZ instead of CNOT gates is missing. | Added justification explaining CZ gates’ symmetry, hardware compatibility, and equivalent entangling capability. | Section 3.6 – Variational Quantum Classifier (VQC) | R2.8 |
| 9. Abbreviation usage is inconsistent. | Ensured all abbreviations are defined once and used consistently throughout the manuscript. | Throughout the manuscript | R2.9 |
| 10. Subscripts for i-th instance are improperly represented (Eqs. 14–16). | Removed problematic equations and corrected notation in remaining equations. | Sections 3.6 | R2.10 |
| 11. Equation 15 is incorrectly printed. | Removed Equation 15 as part of unifying the loss formulation. | NA | NA |
| 12. Some equations are not aligned with their subsections. | Reorganized equations to appear only in their appropriate methodological subsections. | Sections 3.5–3.7 | R2.12 |
| 13. Subsection 3.7 is unclear. | Completely rewrote the subsection | Section 3.7 – Evaluation Setup and Metrics, now it is 3.8 | R2.13 |
| 14. Dataset size, split ratio, and class balance are missing. | Added a “Dataset Composition and Split” paragraph describing simulated vs. real data, SLG/DL/TP/HIF balance, and a 70/15/15 split corresponding to results. | Section 3.3 – Data Preprocessing and Feature Normalization | R2.14 |
| 15. Confusion matrix axes are unclear. | Updated the confusion matrix caption to explicitly define x- and y-axis meanings. | After Figure 4 caption | R2.15 |
| 16. Training and validation curves (Figs. 8–9) are unclear. | Clarified that training and validation loss and accuracy curves are presented in Figure 6 and correspond to the proposed HQCNN model. | Figure 6 caption | R2.16 |
| 17. Permissions for smart grid monitoring data are unclear. | Clarified that all PMU/SCADA data were obtained from publicly accessible repositories and added a Data Availability Statement. | Section 3.2; Data Availability Statement | R2.17 |
| 18. Meaning of ablation study and tuned hyperparameters is unclear. | Explained that the ablation study removes the quantum layer while keeping all other settings unchanged and that no hyperparameters were tuned. | Section 4.5 – Ablation Study | R2.18 |
| 19. What form of error correction is implemented? | Clarified that only measurement (readout) error mitigation using Qiskit Ignis is applied and no full quantum error correction codes are used. | Section 3.6 – Variational Quantum Classifier (VQC) | R2.19 |
| 20. Can HQCNN detect multiple faults? | Clarified that the model is evaluated for single-fault classification only and that multi-fault detection is left for future work. | Section 5 – Conclusion and Future Work | R2.20 |
| 21. Justification of the “high-dimensional entanglement” claim. | Rephrased the statement to avoid overclaiming physical quantum advantage and attributed gains to improved modeling of non-linear feature interactions. | Section 4.1 – Performance on Simulated Data | R2.21 |
