# Supplementary material for: Hybrid quantum-classical neural networks for real-time fault detection in power systems
Source: PLoS One. 2026 Jun 18;21(6):e0349887. doi: 10.1371/journal.pone.0349887 (PMC13278437; doi:10.1371/journal.pone.0349887)
Supplement: Peer review history

# Peer review: Decision Letter 0

**DOI:** 10.1371/journal.pone.0349887.r001

**Authors:** Palaniyappan Sathyaprakash

17 Dec 2025

PONE-D-25-43455

Hybrid Quantum-Classical Neural Networks for Real-Time Fault Detection in Power Systems

PLOS One

Dear Dr. Massebo,

Thank you for submitting your manuscript to PLOS ONE. After careful consideration, we feel that it has merit but does not fully meet PLOS ONE’s publication criteria as it currently stands. Therefore, we invite you to submit a revised version of the manuscript that addresses the points raised during the review process.

Please submit your revised manuscript by Jan 31 2026 11:59PM. If you will need more time than this to complete your revisions, please reply to this message or contact the journal office at plosone@plos.org. When you're ready to submit your revision, log on to https://www.editorialmanager.com/pone/ and select the 'Submissions Needing Revision' folder to locate your manuscript file.

Please include the following items when submitting your revised manuscript:

- A letter that responds to each point raised by the academic editor and reviewer(s). You should upload this letter as a separate file labeled 'Response to Reviewers'.
- A marked-up copy of your manuscript that highlights changes made to the original version. You should upload this as a separate file labeled 'Revised Manuscript with Track Changes'.
- An unmarked version of your revised paper without tracked changes. You should upload this as a separate file labeled 'Manuscript'.

-->If you would like to make changes to your financial disclosure, please include your updated statement in your cover letter. Guidelines for resubmitting your figure files are available below the reviewer comments at the end of this letter.

If applicable, we recommend that you deposit your laboratory protocols in protocols.io to enhance the reproducibility of your results. Protocols.io assigns your protocol its own identifier (DOI) so that it can be cited independently in the future. For instructions see: https://journals.plos.org/plosone/s/submission-guidelines#loc-laboratory-protocols. Additionally, PLOS ONE offers an option for publishing peer-reviewed Lab Protocol articles, which describe protocols hosted on protocols.io. Read more information on sharing protocols at https://plos.org/protocols?utm_medium=editorial-email&utm_source=authorletters&utm_campaign=protocols.

We look forward to receiving your revised manuscript.

Kind regards,

Palaniyappan Sathyaprakash, Ph.D

Academic Editor

PLOS One

Journal Requirements:

When submitting your revision, we need you to address these additional requirements.

1. Please ensure that your manuscript meets PLOS ONE's style requirements, including those for file naming. The PLOS ONE style templates can be found at

https://journals.plos.org/plosone/s/file?id=wjVg/PLOSOne_formatting_sample_main_body.pdf and

https://journals.plos.org/plosone/s/file?id=ba62/PLOSOne_formatting_sample_title_authors_affiliations.pdf

2. Please note that PLOS One has specific guidelines on code sharing for submissions in which author-generated code underpins the findings in the manuscript. In these cases, we expect all author-generated code to be made available without restrictions upon publication of the work. Please review our guidelines at https://journals.plos.org/plosone/s/materials-and-software-sharing#loc-sharing-code and ensure that your code is shared in a way that follows best practice and facilitates reproducibility and reuse.

3. Please update your submission to use the PLOS LaTeX template. The template and more information on our requirements for LaTeX submissions can be found at http://journals.plos.org/plosone/s/latex.

4. Please provide a complete Data Availability Statement in the submission form, ensuring you include all necessary access information or a reason for why you are unable to make your data freely accessible. If your research concerns only data provided within your submission, please write "All data are in the manuscript and/or supporting information files" as your Data Availability Statement.

5. PLOS requires an ORCID iD for the corresponding author in Editorial Manager on papers submitted after December 6th, 2016. Please ensure that you have an ORCID iD and that it is validated in Editorial Manager. To do this, go to ‘Update my Information’ (in the upper left-hand corner of the main menu), and click on the Fetch/Validate link next to the ORCID field. This will take you to the ORCID site and allow you to create a new iD or authenticate a pre-existing iD in Editorial Manager.

6. Please upload a new copy of Figures 1 and 2 as the detail is not clear. Please follow the link for more information:  https://journals.plos.org/plosone/s/figures

7. We note you have included a table to which you do not refer in the text of your manuscript. Please ensure that you refer to Tables 1 and 2 in your text; if accepted, production will need this reference to link the reader to the Table.

8. If the reviewer comments include a recommendation to cite specific previously published works, please review and evaluate these publications to determine whether they are relevant and should be cited. There is no requirement to cite these works unless the editor has indicated otherwise.

9. Please review your reference list to ensure that it is complete and correct. If you have cited papers that have been retracted, please include the rationale for doing so in the manuscript text, or remove these references and replace them with relevant current references. Any changes to the reference list should be mentioned in the rebuttal letter that accompanies your revised manuscript. If you need to cite a retracted article, indicate the article’s retracted status in the References list and also include a citation and full reference for the retraction notice.

[Note: HTML markup is below. Please do not edit.]

Reviewers' comments:

Reviewer's Responses to Questions

**Comments to the Author**

1. Is the manuscript technically sound, and do the data support the conclusions?

The manuscript must describe a technically sound piece of scientific research with data that supports the conclusions. Experiments must have been conducted rigorously, with appropriate controls, replication, and sample sizes. The conclusions must be drawn appropriately based on the data presented.

Reviewer #1: Partly

Reviewer #2: Yes

**********

2. Has the statistical analysis been performed appropriately and rigorously?

Reviewer #1: Yes

Reviewer #2: Yes

**********

3. Have the authors made all data underlying the findings in their manuscript fully available?

The PLOS Data policy requires authors to make all data underlying the findings described in their manuscript fully available without restriction, with rare exception (please refer to the Data Availability Statement in the manuscript PDF file). The data should be provided as part of the manuscript or its supporting information, or deposited to a public repository. For example, in addition to summary statistics, the data points behind means, medians and variance measures should be available. If there are restrictions on publicly sharing data—e.g. participant privacy or use of data from a third party—those must be specified.

Reviewer #1: No

Reviewer #2: No

**********

4. Is the manuscript presented in an intelligible fashion and written in standard English?

PLOS ONE does not copyedit accepted manuscripts, so the language in submitted articles must be clear, correct, and unambiguous. Any typographical or grammatical errors should be corrected at revision, so please note any specific errors here.

Reviewer #1: Yes

Reviewer #2: Yes

**********

5. Review Comments to the Author

Please use the space provided to explain your answers to the questions above. You may also include additional comments for the author, including concerns about dual publication, research ethics, or publication ethics. (Please upload your review as an attachment if it exceeds 20,000 characters)

Reviewer #1: I have reviewed the manuscript and it presents a great use case for fault detection in power systems . The methods are simple and replicable and is practical in NISQ devices. How ever I have some minor concerns related to encoding , VQC and Use of optimizers .

Reviewer #2: 1. The purpose of Table 3, 4, and 5 and their corresponding columns is not very clear.

2. In Table 4, the meaning or measure of the various entries under the column 'classical techniques' and 'deep learning' is not very clear. What does moderate, high and other keywords stand for and how is this measured?

3. In Table 5 lists the various datasets, are these dataset overlapping? Their names if any, needs to be specified.

4. Font size of Fig1, Fig2 needs to be increased as they are not readable

5. The Figure 3 can be accommodated as a whole.

6. The representation of the variables in different equation should be consistent for a given variable. Eqn 1 and eqn 2 has different representation for the input signal.

7. In Fig5, the role of ‘even synchronization’ and ‘missing data handling’ is not clear. The paper begins with taking a signal and cutting it into parts. Request you to justify the missing data.

8. In VQC, why have you used CZ gates for entanglement and why not CNOT gate? Justification for the same is needed.

9. If you are using abbreviations for a particular word then be consistent and use the same throughout the paper. There is no need to use both the full form and abbreviation ahead in the paper.

10. To represent ith instance in an equation use subscript. Eqn 14, 15, and 16 subscripts are not properly represented.

11. I feel Eqn 15 is not correctly printed.

12. Equation 15 and 16 are not a part of the subsection 3.5. Similarly eqn 11, 12 and 13 are not related to subsection 3.4

13. Subsection 3.7 needs to be rewritten as it not very clear.

14. For Fig 6. and Table 7 what is the split ratio? How many samples were used for training and testing? Subsection 3.2 speaks about the data gathering but doesn’t tell us how many samples were simulated and how many of them actually collected and total count in terms of (SLG), double-line (DL), and three-phase (TP) faults. This information might is very much nesseary to see if the dataset is balanced.

15. What does the X and Y labels of confusion matrix stand for?

16. Fig. 8 and 9 shows training and validation accuracies/loss using for which particular techniques?

17. You are using the data from the project 'smart grid monitoring project' are proper permissions sought from the concerned authority?

18. In subsection 4.5, please explain the meaning of the 'An ablation study was conducted to assess the individual contribution of the quantum layer'. What hyper parameter was tuned in this ablation study?

19. This work speaks about applying error correction, what form of error corrections is implemented?

20. Can your proposed techniques of HQCNN detect multiple faults? If Yes, a justification needs to be given in the form of results comparing the various possible combinations.

21. Can you justify this statement used in your paper, 'The performance gain is attributed to the ability of the quantum layer to capture high-dimensional entanglements in the feature space, enhancing decision boundaries for ambiguous cases'.

**********

6. PLOS authors have the option to publish the peer review history of their article (what does this mean?). If published, this will include your full peer review and any attached files.

If you choose “no”, your identity will remain anonymous but your review may still be made public.

**Do you want your identity to be public for this peer review?**    For information about this choice, including consent withdrawal, please see our Privacy Policy.

Reviewer #1: No

Reviewer #2: No

**********

[NOTE: If reviewer comments were submitted as an attachment file, they will be attached to this email and accessible via the submission site. Please log into your account, locate the manuscript record, and check for the action link "View Attachments". If this link does not appear, there are no attachment files.]

To ensure your figures meet our technical requirements, please review our figure guidelines: https://journals.plos.org/plosone/s/figures

You may also use PLOS’s free figure tool, NAAS, to help you prepare publication quality figures: https://journals.plos.org/plosone/s/figures#loc-tools-for-figure-preparation.

NAAS will assess whether your figures meet our technical requirements by comparing each figure against our figure specifications.

Attachment Submitted filename: Manuscript_Review_Normalisation_VQC.docx

Attachment Submitted filename: PONE-D-25-43455.docx

# Author comment: Author response to Decision Letter 1

**DOI:** 10.1371/journal.pone.0349887.r002

11 Feb 2026

Manuscript ID: PONE-D-25-43455

Title: Hybrid Quantum-Classical Neural Networks for Real-Time Fault Detection in Power Systems Response to Reviewer #1

Reviewer #1 Comment Action Taken / Response Location of Change in Manuscript Reviewer Comment ID (used in manuscript)

1. Clarify whether normalization is performed per window or across the dataset. Clarified that Min–Max normalization parameters are computed using only the training set and then applied unchanged to validation and test sets to avoid information leakage and preserve amplitude characteristics. Section 3.3 – Data Preprocessing and Feature Normalization R1.1

2. Angle encoding limits expressivity due to restricted rotation range. Updated the encoding scheme to a centered angle mapping to preserve sign information and enhance expressivity for shallow variational quantum circuits. Section 3.5 – Classical-to-Quantum Encoding, Equation (8) R1.2

3. Quantum circuit figure shows fixed rotation angles instead of trainable parameters. Redrew the quantum circuit to explicitly depict trainable rotation gates (R_y(\phi_j^{(\ell)})) for all qubits and layers, ensuring consistency between text and figure. Figure 3, Section 3.6 – Variational Quantum Classifier (VQC) R1.3

4. Optimizer choice for quantum parameters is unclear. Explicitly stated that the SPSA optimizer is used exclusively for training quantum parameters due to its robustness in noisy NISQ environments. Sections 3.6 and 3.7 R1.4

5. Multiple or inconsistent loss formulations appear in the manuscript. Unified the training objective by defining a single loss formulation and removing redundant loss equations, ensuring consistency across all sections. Section 3.7 – Hybrid Training and Optimization Strategy R1.5

6. Error correction claims may be overstated. Clarified that only measurement (readout) error mitigation using Qiskit Ignis is applied, and no full quantum error correction codes are implemented. Section 3.6 – Variational Quantum Classifier (VQC) R1.6

7. Possible train–test leakage due to window-based segmentation. Added an explicit statement confirming that data splitting is performed at the fault-event level so that all windows from a given event belong exclusively to one dataset split. Section 3.2 – Data Collection and Preprocessing R1.7

Response to Reviewer #2

Reviewer #2 Comments Action Taken / Response Location of Change in Manuscript Reviewer Comment ID (used in manuscript)

1. Purpose of Tables 3–5 is unclear. Added an explanatory paragraph describing the intent and scope of Tables 3–5. Section 2.4 Paragraph immediately preceding Tables 3–5 R2.1

2. Qualitative terms in Table 4 (High, Moderate, etc.) are unclear. Added a footnote defining all qualitative descriptors based on reported empirical evidence. Section 2.4 Table 4 footnote R2.2

3. Dataset overlap in Table 5 is unclear. Added an explicit “Overlap” column, clarified non-overlapping datasets, and included a row describing the dataset used in this work. Table 5; Section 3.2 R2.3

4. Figures 1 and 2 are not readable. Regenerated Figures 1 and 2 with increased font size and improved resolution. Figures 1 and 2 R2.4

5. Figure 3 should be presented as a single figure. Redrew Figure 3 as a unified four-qubit, three-layer variational quantum circuit with corrected labels. Figure 3 R2.5

6. Inconsistent variable notation across equations. Unified notation across all equations using consistent symbols for inputs, encoding, and quantum operations. Sections 3.1–3.5 R2.6

7. Role of missing data handling is unclear. Expanded preprocessing description to include synchronization, denoising, and linear interpolation for short missing segments. Section 3.2 R2.7

8. Justification for using CZ instead of CNOT gates is missing. Added justification explaining CZ gates’ symmetry, hardware compatibility, and equivalent entangling capability. Section 3.6 – Variational Quantum Classifier (VQC) R2.8

9. Abbreviation usage is inconsistent. Ensured all abbreviations are defined once and used consistently throughout the manuscript. Throughout the manuscript R2.9

10. Subscripts for i-th instance are improperly represented (Eqs. 14–16). Removed problematic equations and corrected notation in remaining equations. Sections 3.6 R2.10

11. Equation 15 is incorrectly printed. Removed Equation 15 as part of unifying the loss formulation. NA NA

12. Some equations are not aligned with their subsections. Reorganized equations to appear only in their appropriate methodological subsections. Sections 3.5–3.7 R2.12

13. Subsection 3.7 is unclear. Completely rewrote the subsection Section 3.7 – Evaluation Setup and Metrics, now it is 3.8 R2.13

14. Dataset size, split ratio, and class balance are missing. Added a “Dataset Composition and Split” paragraph describing simulated vs. real data, SLG/DL/TP/HIF balance, and a 70/15/15 split corresponding to results. Section 3.3 – Data Preprocessing and Feature Normalization R2.14

15. Confusion matrix axes are unclear. Updated the confusion matrix caption to explicitly define x- and y-axis meanings. After Figure 4 caption R2.15

16. Training and validation curves (Figs. 8–9) are unclear. Clarified that training and validation loss and accuracy curves are presented in Figure 6 and correspond to the proposed HQCNN model. Figure 6 caption R2.16

17. Permissions for smart grid monitoring data are unclear. Clarified that all PMU/SCADA data were obtained from publicly accessible repositories and added a Data Availability Statement. Section 3.2; Data Availability Statement R2.17

18. Meaning of ablation study and tuned hyperparameters is unclear. Explained that the ablation study removes the quantum layer while keeping all other settings unchanged and that no hyperparameters were tuned. Section 4.5 – Ablation Study R2.18

19. What form of error correction is implemented? Clarified that only measurement (readout) error mitigation using Qiskit Ignis is applied and no full quantum error correction codes are used. Section 3.6 – Variational Quantum Classifier (VQC) R2.19

20. Can HQCNN detect multiple faults? Clarified that the model is evaluated for single-fault classification only and that multi-fault detection is left for future work. Section 5 – Conclusion and Future Work R2.20

21. Justification of the “high-dimensional entanglement” claim. Rephrased the statement to avoid overclaiming physical quantum advantage and attributed gains to improved modeling of non-linear feature interactions. Section 4.1 – Performance on Simulated Data R2.21

Attachment Submitted filename: Response_to_Reviewers.docx

# Peer review: Decision Letter 1

**DOI:** 10.1371/journal.pone.0349887.r003

**Authors:** Palaniyappan Sathyaprakash

6 May 2026

Hybrid Quantum-Classical Neural Networks for Real-Time Fault Detection in Power Systems

PONE-D-25-43455R1

Dear Dr. Massebo,

We’re pleased to inform you that your manuscript has been judged scientifically suitable for publication and will be formally accepted for publication once it meets all outstanding technical requirements.

Within one week, you’ll receive an e-mail detailing the required amendments. When these have been addressed, you’ll receive a formal acceptance letter and your manuscript will be scheduled for publication.

An invoice will be generated when your article is formally accepted. Please note, if your institution has a publishing partnership with PLOS and your article meets the relevant criteria, all or part of your publication costs will be covered. Please make sure your user information is up-to-date by logging into Editorial Manager at Editorial Manager® and clicking the ‘Update My Information' link at the top of the page. For questions related to billing, please contact billing support.

If your institution or institutions have a press office, please notify them about your upcoming paper to help maximize its impact. If they’ll be preparing press materials, please inform our press team as soon as possible -- no later than 48 hours after receiving the formal acceptance. Your manuscript will remain under strict press embargo until 2 pm Eastern Time on the date of publication. For more information, please contact onepress@plos.org.

Kind regards,

Palaniyappan Sathyaprakash, Ph.D

Academic Editor

PLOS One

Additional Editor Comments (optional):

Reviewers' comments:

Reviewer's Responses to Questions

**Comments to the Author**

1. If the authors have adequately addressed your comments raised in a previous round of review and you feel that this manuscript is now acceptable for publication, you may indicate that here to bypass the “Comments to the Author” section, enter your conflict of interest statement in the “Confidential to Editor” section, and submit your "Accept" recommendation.

Reviewer #1: All comments have been addressed

Reviewer #2: All comments have been addressed

**********

2. Is the manuscript technically sound, and do the data support the conclusions?

The manuscript must describe a technically sound piece of scientific research with data that supports the conclusions. Experiments must have been conducted rigorously, with appropriate controls, replication, and sample sizes. The conclusions must be drawn appropriately based on the data presented.

Reviewer #1: Yes

Reviewer #2: Yes

**********

3. Has the statistical analysis been performed appropriately and rigorously?

Reviewer #1: Yes

Reviewer #2: Yes

**********

4. Have the authors made all data underlying the findings in their manuscript fully available?

The PLOS Data policy requires authors to make all data underlying the findings described in their manuscript fully available without restriction, with rare exception (please refer to the Data Availability Statement in the manuscript PDF file). The data should be provided as part of the manuscript or its supporting information, or deposited to a public repository. For example, in addition to summary statistics, the data points behind means, medians and variance measures should be available. If there are restrictions on publicly sharing data—e.g. participant privacy or use of data from a third party—those must be specified.

Reviewer #1: Yes

Reviewer #2: Yes

**********

5. Is the manuscript presented in an intelligible fashion and written in standard English?

PLOS ONE does not copyedit accepted manuscripts, so the language in submitted articles must be clear, correct, and unambiguous. Any typographical or grammatical errors should be corrected at revision, so please note any specific errors here.

Reviewer #1: Yes

Reviewer #2: Yes

**********

6. Review Comments to the Author

Please use the space provided to explain your answers to the questions above. You may also include additional comments for the author, including concerns about dual publication, research ethics, or publication ethics. (Please upload your review as an attachment if it exceeds 20,000 characters)

Reviewer #1: General Assessment

The revised manuscript presents a relevant and timely study on hybrid quantum-

classical learning for real-time fault detection in power systems. The topic is

suitable for the journal’s scope, and the paper has improved substantially in

response to the previous round of review. In particular, the revised version

is stronger in methodological transparency, notation, and presentation of the

hybrid HQCNN framework. The inclusion of both simulated IEEE test-system

data and real PMU/SCADA events also strengthens the applied relevance of

the study.

Overall, I find that the major technical concerns raised previously, espe-

cially those associated with Reviewer 1, have been addressed adequately. The

manuscript is now close to publishable form. However, a small number of mi-

nor issues remain, primarily related to consistency, language polishing, and a

few residual presentation ambiguities. I therefore recommend acceptance after

minor revision.

Assessment of Reviewer 1 Comments

1. Normalization protocol: addressed. The revised manuscript now

clarifies that Min–Max normalization parameters are computed from the

training set and then applied unchanged to validation and test data. It

also explicitly states that normalization is not performed on a per-window

basis. This satisfactorily resolves the earlier concern regarding possible

information leakage.

2. Expressivity of the angle encoding: addressed. The authors revised

the encoding description and now introduce a centered angle mapping in

Eq. (8), which is a meaningful improvement over the earlier restricted

formulation.

3. Quantum circuit trainable parameters: addressed. Figure 3 and

the corresponding text now indicate trainable rotation parameters, which

aligns the figure with the method description and resolves the earlier in-

consistency.

4. Optimizer specification: addressed. The manuscript now explicitly

states that SPSA is used to optimize the quantum parameters. This addi-

tion improves reproducibility and is appropriate for noisy/NISQ-oriented

workflows.

5. Loss formulation: addressed. The training objective has been consol-

idated into a single formulation, which improves internal consistency and

readability.

6. Error correction claim: addressed. The revised text now correctly

limits the claim to measurement/readout error mitigation using Qiskit

Ignis and no longer overstates the use of full quantum error correction.

7. Train–test leakage via windowing: addressed. The manuscript now

states that splitting is performed at the fault-event level so that all win-

dows derived from a given event remain in a single split. This is an

important clarification and satisfactorily addresses the leakage concern.

Remaining Minor Comments

1. Author name consistency (pages 1 and 8). The corresponding

author name is inconsistent between the submission metadata and the

manuscript body. One location lists Yaikob Abriham Massebo, while the

manuscript title page lists Yakob Abraham. This should be harmonized

before final publication.

2. Abstract language and terminology (page 8). The abstract still

requires a final language polish. Examples include:

• “IEEE 14 and 39-bus system” should be revised to “IEEE 14- and

39-bus systems”;

• “Various kind of fault like SDL” should be revised to “Various kinds

of faults such as SLG”;

• “PMD data sets” appears to be a typo and likely should read “PMU

datasets”.

These are minor editorial issues, but they affect professionalism and clar-

ity.

3. Introduction requires copyediting (pages 8–10). The introduction

has improved conceptually, but several sentences remain grammatically

awkward or stylistically non-standard for a journal article. This applies

especially to the opening paragraph and the subsections “Quantum Com-

puting: A New Paradigm,” “Hybrid Quantum Classical Neural Networks,”

and “Paper Structuring.” A final English-language edit is recommended.

4. Experimental split description should be made fully unambigu-

ous (pages 18–19). Section 3.2 states that simulated data were used

for training and controlled evaluation, while validation and testing were

carried out using real-world events only. However, Section 3.3 states that

the combined dataset was divided into 70/15/15 for training, validation,

and testing. These statements may be reconcilable, but in the current

wording they create confusion. The authors should present one clear and

consistent description of the final experimental protocol.

5. Encoding equations should be checked for internal consistency

(page 20). Eq. (8) introduces a centered encoding, θi = π(2zi − 1), but

Eq. (9) appears to revert to a non-centered form using RY (πzi). If this is

not intentional, the equations should be aligned so that the mathematical

description matches the stated encoding strategy.

6. Duplicate entry in Table 3 (page 13). Table 3 appears to repeat the

row for Ref. [10] (Neural Networks). This duplication should be removed.

7. Data availability statement should be finalized carefully (page 6

and main text). The manuscript states that the data are fully available

and points to a public dataset link. Before final acceptance, the authors

should ensure that the final published manuscript contains a complete

and durable data availability statement in the journal’s preferred format,

including a stable repository link or DOI where applicable.

8. Minor terminology polishing throughout. Although the abbrevia-

tion issue has largely been improved, the manuscript would still benefit

from one final pass to ensure fully consistent usage of terms such as SLG,

DL, TP, HIF, PMU, SCADA, HQCNN, and VQC throughout the text,

tables, and figure captions.

Conclusion

In summary, this is a stronger and more coherent revision. The principal

methodological concerns from the prior review round, especially those raised

by Reviewer 1, have been addressed satisfactorily. The remaining issues are

minor and editorial in nature, with one methodological clarification still needed

regarding the exact train/validation/test protocol. Subject to those small revi-

sions, I support publication.

Final Recommendation: Minor Revision

Reviewer #2: (No Response)

**********

7. PLOS authors have the option to publish the peer review history of their article (what does this mean?). If published, this will include your full peer review and any attached files.

If you choose “no”, your identity will remain anonymous but your review may still be made public.

**Do you want your identity to be public for this peer review?**    For information about this choice, including consent withdrawal, please see our Privacy Policy.

Reviewer #1: No

Reviewer #2: No

**********

# Editor report: Acceptance letter

**DOI:** 10.1371/journal.pone.0349887.r004

**Authors:** Palaniyappan Sathyaprakash

PONE-D-25-43455R1

PLOS One

Dear Dr. Massebo,

I'm pleased to inform you that your manuscript has been deemed suitable for publication in PLOS One. Congratulations! Your manuscript is now being handed over to our production team.

At this stage, our production department will prepare your paper for publication. This includes ensuring the following:

* All references, tables, and figures are properly cited

* All relevant supporting information is included in the manuscript submission,

* There are no issues that prevent the paper from being properly typeset

You will receive further instructions from the production team, including instructions on how to review your proof when it is ready. Please keep in mind that we are working through a large volume of accepted articles, so please give us a few days to review your paper and let you know the next and final steps.

Lastly, if your institution or institutions have a press office, please let them know about your upcoming paper now to help maximize its impact. If they'll be preparing press materials, please inform our press team within the next 48 hours. Your manuscript will remain under strict press embargo until 2 pm Eastern Time on the date of publication. For more information, please contact onepress@plos.org.

You will receive an invoice from PLOS for your publication fee after your manuscript has reached the completed accept phase. If you receive an email requesting payment before acceptance or for any other service, this may be a phishing scheme. Learn how to identify phishing emails and protect your accounts at https://explore.plos.org/phishing.

If we can help with anything else, please email us at customercare@plos.org.

Thank you for submitting your work to PLOS ONE and supporting open access.

Kind regards,

PLOS ONE Editorial Office Staff

on behalf of

Dr. Palaniyappan Sathyaprakash

Academic Editor

PLOS One
